# Aluminum Genotoxicity in Plants, Mammals, and Unicellular Eukaryotes: The Underlying Mechanisms

**DOI:** 10.3390/ijms27041665

**Published:** 2026-02-09

**Authors:** Hossein Zakariapour Bahnamiri, Alica Navrátilová, Marek Kovár, Lucia Klongová, Miroslava Požgajová

**Affiliations:** 1 AgroBioTech Research Center, Slovak University of Agriculture, Tr. A. Hlinku 2, 949 76 Nitra, Slovakia; hossein.bahnamiri@uniag.sk (H.Z.B.); lucia.klongova@uniag.sk (L.K.); 2 Institute of Nutrition and Genomics, Slovak University of Agriculture, Tr. A. Hlinku 2, 949 76 Nitra, Slovakia; alica.navratilova@uniag.sk; 3 Institute of Plant and Environmental Sciences, Slovak University of Agriculture, Tr. A. Hlinku 2, 949 76 Nitra, Slovakia; marek.kovar@uniag.sk

**Keywords:** aluminum toxicity, mammalian cells, genotoxicity, chromosome aberrations, cell division, cell death

## Abstract

Aluminum (Al), the third most abundant metal in the Earth’s crust, has been preserved for thousands or even millions of years. However, acidic rain and soil acidification, largely driven by human activities related to industrialization and the increased use of Al in daily life, have led to the mobilization of Al from its complex natural resources. This exposure has affected various microorganisms, including bacteria, fungi, protozoa, and yeast, as well as macroorganisms such as plants, animals, and humans, by introducing them to Al in its ionic form. To date, no biological role for Al has been defined in organisms; however, some beneficial effects have been shown, particularly on plant growth. The exposure of living organisms, particularly human cell lines, to chronic and high doses of Al has been the focus of numerous studies. The consequences of such exposure can vary significantly based on the type of organism, their sensitivity, the form of Al, and the dosage used. In plants, these consequences can include inhibited root growth, stunted development, reduced biomass, and disrupted nutrient uptake. In animals, Al exposure can lead to neurological impairments, impaired mineral metabolism, and bone abnormalities. In humans, it may result in encephalopathy, cognitive deficits, microcytic anemia, and an increased risk of Alzheimer’s disease. Unicellular organisms, such as yeast and bacteria, may experience decreased cell viability, inhibited growth, and disrupted metabolic processes. This review discusses the genotoxicity of Al in plants, mammals, and yeast cells, as well as the subsequent detrimental effects on cell cycle and cell proliferation. It also explores the underlying mechanisms and pathways associated with these effects. Furthermore, the types of Al-induced cell death as a response mechanism to Al toxicity and the pathways involved in various cell types were discussed.

## 1. Introduction

Aluminum (Al) is the third most abundant element in the Earth’s crust, comprising approximately 8% of its composition. In natural environments, Al is primarily found in inert forms such as Al oxides and aluminosilicate minerals within the soil. Under normal conditions, these compounds pose little threat to living organisms. However, environmental disturbances, largely driven by human activities, can lead to their mobilization into bioavailable and toxic forms. Among these triggers, acidification of agricultural soils through acid rain and the use of nitrogen-based fertilizers are particularly significant. It is estimated that over 50% of the world’s arable land consists of acidic soils [1]. When the soil pH drops below 5, Al is converted into phytotoxic species, such as hydroxo- and oxo-aluminum species, accompanied by Al^3+^ release, which readily enter root tip cells and inhibit cell division and elongation, thereby impairing root development. Although the hydrolysis of Al salts, such as AlCl_3_, in acidic soils and aquatic solutions releases HCl, which can further acidify the medium and complicate toxicity assessments, studies show that Al toxicity is not solely due to pH reduction, as using HCl alone does not replicate the cellular and physiological effects seen with Al exposure [2]. Mobilized Al can also leach into freshwater systems, affecting aquatic organisms. Beyond environmental exposure, Al has become increasingly prevalent in human life through its widespread use in cookware, food processing, cosmetics, pharmaceuticals, vaccines, antiperspirants, pesticides, food additives, and even air pollutants [3,4]. As the second most widely used metal after steel, Al plays a major role in industrial applications, including construction, packaging, and electronics [5,6]. The average exposure from Al cookers was reported to be six times higher than the recommended daily intake by the World Health Organization for adults, and the amount of Al absorbed through drug utilization was higher than the amount absorbed via diet and water consumption [7].

Unlike some trace metals, Al has no known beneficial biological role in living organisms. Instead, its accumulation, especially in bioavailable ionic forms such as Al^3+^ and Al(OH)_2_^+^, can lead to harmful effects across a wide range of species. Chronic and acute exposure to Al can cause various dysfunctions in various organisms. As a non-biodegradable contaminant, Al can accumulate in plants, microorganisms, animals, and humans, where it exerts cytotoxic, genotoxic, and mutagenic effects [8]. Al ions are highly reactive classified as class A (oxygen-seeking) cations which preferentially bind to negative donor groups such as carboxyl and phosphate; hence, many biological components and structures, including carboxyl residues of pectin in the cell wall, phospholipids in the plasma membrane, glutamate, and aspartate residues in various proteins and phosphate residues in nucleotide and nucleic acids, can be the potential target of Al ions [9]. A key mechanism underlying Al toxicity is the induction of oxidative stress through excessive reactive oxygen species (ROS) generation, which contributes to cellular damage and death in various organisms. In plants, Al predominantly affects roots, where it causes mitochondrial dysfunction, disrupts plasma membrane integrity through iron-mediated lipid peroxidation, and triggers vacuolar collapse via upregulation of genes such as the vacuolar processing enzyme in tobacco (*Nicotiana tabacum* L.), NtVPE1 [10]. Additionally, studies in Arabidopsis have shown that AlCl_3_ interacts with DNA, potentially via electrostatic binding to the phosphodiester backbone, leading to genomic instability [11,12]. Al can accumulate in all animal tissues, with a preference for the kidneys, liver, heart, bones, and brain; however, bone tissue is the primary site for Al accumulation, comprising more than 50% of the Al in the body [13]. In humans, chronic Al exposure has been linked to a range of disorders, including bone diseases [14], microcytic anemia [15], neurodegenerative conditions [16], and breast cancer [17]. These effects may result from both direct interactions with genetic material and indirect mechanisms such as ROS-mediated damage and impaired cell division. The current review discusses the genotoxic effects of Al across various biological systems, with a focus on its disruption of essential cellular processes such as DNA synthesis, cytoskeletal organization, chromosome dynamics, and cell cycle regulation. Additionally, we discuss Al-induced cell death pathways, including programmed cell death (PCD) types, necrosis, and autophagy, in organisms ranging from plants and yeast to mammals.

## 2. Al Exposure in Plants

### 2.1. Al-Mediated Chromosome Abnormalities in Plant Cells

Dissociation of Al from its salt can alter the ionic environment of the cell, leading to physiological changes in nucleoproteins or protein denaturation, and eventually to chromosomal aberrations. Al toxicity disrupts cell division, characterized by physiological alteration of nucleoproteins and causing chromosomal aberrations, including chromosomal adhesion, chromosomal fragmentation, c-mitosis, and chromosomal bridges, as shown in Masson pine (*Pinus massoniana*) treated with different concentrations of AlCl_3_ (10^−5^–10^−2^ M) for 40 days [18]. Stickiness, as another chromosomal abnormality, can occur due to increased chromosome contraction and condensation, depolymerized DNA, and partial dissolution of nucleoproteins of plants treated with heavy metals, including Al. Al exhibited an inhibitory effect on root growth at concentrations of 50 and 100 μM of Al_2_(SO_4_)_3_ in *Vicia faba* during a 72-h treatment [19]. Chromosome fragmentation, for instance, in Masson pine in response to 10^−5^–10^−2^ M AlCl_3_, may occur due to the over-strengthening of chromosome bridges, which can lead to chromatid breaks at or near the points of these bridges during anaphase [18]. Furthermore, chromosome fragmentation has been suggested as a possible reason for the higher pollen sterility. Other mechanisms of Al-mediated toxicity include an imbalance in phytohormones and the resulting impairment of signal transduction pathways. It has been reported that the exogenous application of phytohormones such as auxin, cytokinin, and abscisic acid can interact with other pathways, thereby regulating plant responses to Al toxicity [20]. In an in situ experiment, single, low-dose (50 µM) and short-term exposure of germinated rice seeds, *Oryza sativa* cv. Lalat to AlCl_3_ caused long-term damage to the plant. Al exposure caused chromosomal stickiness, micronuclei formation, laggards, sticky bridges, and also binucleated and multinucleated cells compared to the control group [21]. The formation of binucleated and multinucleated cells in response to Al was related to the inhibition of cell plate formation. Micronuclei are an indicator of disturbed DNA integrity caused by chromosomal breakage or aneuploidy, and their formation in response to Al^3+^ ions was reported in species such as barley roots, rice, maize, *Vicia faba*, and *Allium sativum* roots [21,22,23,24]. This demonstrates that Al acts both as a clastogen, capable of inducing breaks in chromosomes and chromatids, and as an aneugen, promoting chromosome missegregation such as Al-mediated lagging chromosomes. Moreover, Al-induced ROS attack to purine and pyrimidine bases and deoxyribose in DNA can cause breaks in the DNA single and double strands, leading to enhanced chromosomal aberrations and micronuclei formation [24].

**Figure 1:**
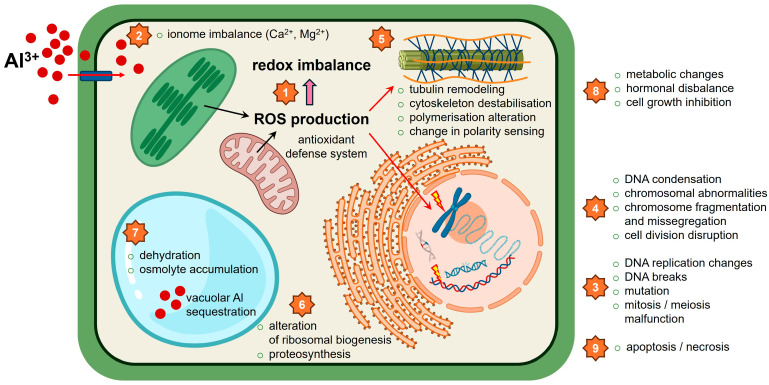
Mechanisms of Aluminum (Al)-induced toxicity in plant cells. Exposure of plants to Al leads to excessive ROS production, overwhelming the antioxidant defense system and causing redox imbalance (1). This oxidative stress subsequently results in ionic imbalance, particularly through impairment of Ca^2+^ and Mg^2+^ homeostasis (2). Al exposure is also associated with DNA damage, leading to mutations and cell cycle disturbances (3), as well as chromosome fragmentation and missegregation during cell division (4). In addition, Al induces cytoskeletal abnormalities through tubulin remodeling, altered polymerization, or disrupted polarity sensing (5). Alterations in protein synthesis and ribosomal biogenesis are commonly observed under Al toxicity (6). As a defense mechanism, plant cells sequester Al into the vacuole for detoxification (7). Al-mediated inhibition of cell growth is linked to metabolic alterations and hormonal imbalance (8), which may ultimately lead to cell death (9).

Chromosome abnormalities mediated by Al exposure in plant cells are summarized in Table 1 and visualized in Figure 1.

### 2.2. Al Effects on the Plant Cell Cytoskeleton

The cytoskeleton is responsible for various cellular processes such as cell growth, differentiation, cell division, and internal arrangement, contributing to root growth. It contains a network of microtubules, actin filaments, and other related proteins, which are potential targets for cytosolic Al toxicity [25,26,27]. Cytoskeleton orientation provides a template for cell division and biosynthesis of the cell wall. Al toxicity can disrupt the structure of the cytoskeleton, which is needed for the proper functioning of cell differentiation and cell division [2,28]. Transcriptomic analyses revealed that exposure to 1 or 1.2 mM AlCl_3_ results in down-regulation of numerous genes involved in cytoskeleton metabolism in Al-intolerant ‘sour pummelo’ (*Citrus grandis*) compared to Al-tolerant ‘Xuegan’ (*Citrus sinensis*) citrus species [29,30]. The mechanisms controlling the organization of the microtubule cytoskeleton and also tubule polymerization can be affected by 1 mM of AlCl_3_ in dividing root-tip cells of *Triticum turgidum*, leading to delayed microtubule disassembly and altered tubule polymerization during mitosis and subsequently impaired chromosome movements by the mitotic spindle and eventually restriction of root growth [31]. The effects can result from direct interactions of Al^3+^ ions with cytoskeletal components, such as microtubules and actin filaments, or indirectly by modifying cytosolic Ca^2+^ levels that are crucial for cytoskeletal stability [32,33]. Organization of both microtubules and microfilaments in root cells can be disrupted by Al exposure [31,34]. Al exposure in the form of AlCl_3_ and concentration ranging from 0.1 to 50 µM also caused a considerable enhancement in actin filament tension in soybean (*Glycine max* L.) root tip cells [35]. When seedling roots of *Zea mays* were treated with 50 µM of AlCl_3_, the orientation of microtubules was considerably altered in protoderm cells and the two outer cortex layers, and interphase cortical microtubule arrays converted from transverse to random and/or longitudinal organization [36]. The altered microtubule orientation resulted in the division plane changing from transverse to longitudinal and led to daughter cells positioned side by side instead of above and below due to Al-induced change in normal polarity sensing mechanism, which was proposed to be regulated by actin filaments. Moreover, as control of actin filament formation is tightly controlled by Ca^2+^, and activities of Ca^2+^-activated actin-binding proteins such as gelsolin [37,38], actin organization can be indirectly affected by Al-induced inhibition of Ca^2+^ homeostasis, leading to altered orientation of microtubules [39,40]. Importantly, calmodulin is an important receptor linking changes in Ca^2+^ levels with cytoskeletal functions [41]. Moreover, exposure to Al (80 or 400 µM of AlCl_3_) was reported to affect the physical characteristics of actin filaments in Al-sensitive wheat (*Triticum aestivum* cv. Victory), leading to fimbrin-like protein (an actin bundling protein) mRNA accumulation. Fimbrin was thus reported to be involved in actin organization and proper cell division regulation, as accumulation of its transcripts is associated with alterations of cytoskeletal function maintenance [42]. During the response of plants to stress, in addition to the disruption of ROS homeostasis, tubulin cytoskeleton remodeling plays a key role in the acquisition of mitogen-activated protein kinase via MAPK signaling cascade and the regulatory mechanisms both at the transcriptional and post-transcriptional levels [43,44,45]. The complete or partial depolymerization of microtubules, along with the formation of atypical tubulin structures such as macrotubules and paracrystalline tubulin strands, has been associated with excessive production of ROS in response to oxidative stress in *Triticum turgidum* and *Arabidopsis thaliana* [46]. This oxidative stress is caused by abiotic factors and may also be linked to Al toxicity.

Al binds nucleoside triphosphates with an affinity nearly 107 times greater than Mg^2+^, and Al^3+^-ATP/GTP complexes hydrolyze about 105 times more slowly than their Mg^2+^ counterparts, leading to rapid displacement of Mg^2+^ in nucleotide complexes [47,48]. Actin and tubulin dynamics and microfilament and microtubule stability/instability are respectively regulated by binding and hydrolysis of Mg^2+^-ATP and Mg^2+^-GTP complexes. Hence, altered kinetics of these reactions can lead to disrupted organization of the cytoskeleton network and impair growth-related activities such as cell division and pollen-tube elongation in soybean (*Glycine max*) root cells [35]. The actin cytoskeleton and microtubules are physically linked to the nuclear envelope, helping maintain nuclear shape, nucleolar positioning, and chromatin organization, including nucleolar organizer regions (NORs) [49,50]. Al disrupts the plant cytoskeletal dynamics (e.g., by its interference with Mg^2+^-ATP/GTP-dependent actin and tubulin function), and because cytoskeletal integrity is required for proper nuclear, nucleolar, and NORs organization, this disruption might indirectly contribute to NOR fragmentation, persistence, and extrusion.

NORs are key nucleolar structures that contain ribosomal RNA (rRNA) genes essential for ribosome biogenesis, protein synthesis, and normal cellular function. In several plant species, Al stress disrupts NOR structure and localization. In *Vicia faba* L. root tip cells, and in *Pinus massoniana*, AlCl_3_ treatment caused fragmentation and dispersion of NORs within the nucleus. Prolonged Al exposure (24–72 h) and increasing concentrations (10–10,000 µM) further promoted the extrusion of NORs into the cytoplasm [18,51]. Similar NOR extrusion was observed in *Allium cepa* roots treated with 50 µM AlCl_3_ [52] and in *Allium sativum* exposed to 10–100,000 µM AlCl_3_ [53]. This abnormal relocation of NORs may result from oxidative damage to proteins of the nuclear pore complex (NPC), which regulates nucleolar-nuclear transport [54]. Under normal conditions, nucleoli disassemble during mitosis; therefore, the presence of persistent nucleoli indicates altered nucleolar dynamics and increased nucleolar activity [55,56]. In *Allium cepa* roots, the increased frequency of persistent nucleoli following AlCl_3_ treatment (50 µM) appears to represent an adaptive response to Al-induced stress [52]. Additionally, Al-induced oxidative stress and excessive ROS production can cause cleavage of nucleolin, a major structural protein of the nucleolus, further contributing to nucleolar dysfunction [57].

The effects of Al exposure on the plant cell cytoskeleton are summarized in Table 1 and graphically depicted in Figure 1.

### 2.3. Al Toxicity and the Plant Cell Cycle

Al^3+^ ions are known to directly damage DNA, and studies in *Arabidopsis thaliana* have shown that the DNA damage response (DDR) pathway plays a central role in plant responses to Al stress. Activation of the DDR leads to a temporary arrest of the cell cycle and initiates DNA repair processes [58]. Several genes are critical for this response, including ATR (Ataxia Telangiectasia and Rad3-related), ATM (Ataxia Telangiectasia Mutated), SOG1 (Suppressor of Gamma Response 1), ALT2 (Al Tolerant 2), and SUV2. ATR and ATM are serine/threonine protein kinases that function as key DNA damage sensors. ATR is primarily activated by persistent single-stranded DNA (ssDNA), whereas ATM responds to DNA double-strand breaks (DSBs). Upon activation, both kinases phosphorylate SOG1, a central DDR transcription factor that regulates the expression of hundreds of genes involved in DNA repair and cell cycle inhibition. ALT2, a WD-40 repeat protein, contributes to DNA integrity surveillance, including the detection of DNA crosslinks, while SUV2 encodes a putative ATR-interacting protein that facilitates ATR recruitment to damaged DNA. Together, these components coordinate cell cycle arrest following Al exposure in *Arabidopsis* [59]. Due to its high reactivity, Al can interact with multiple cellular structures, including the cell wall, plasma membrane, cytoskeleton, and nucleus. The most severe toxic effects occur in the distal transition zone of the root tip, where meristematic cells shift from active division to F-actin-dependent elongation [60,61]. In maize, exposure to Al^3+^ (50 µM AlCl_3_ with 200 µM CaCl_2_) inhibits both cell division in the meristematic zone and cell elongation in the elongation zone. A major contributing factor to this inhibition is disrupted calcium (Ca^2+^) uptake, which directly and indirectly impairs cell cycle progression and root elongation [62].

While activation of the DDR pathway represents a regulated mechanism by which plants temporarily halt the cell cycle to repair Al-induced DNA lesions, Al also exerts direct physicochemical effects on nuclear components that further disrupt cell cycle progression. These direct interactions with chromatin, DNA, and nuclear transport machinery amplify DDR-mediated growth inhibition and result in widespread cytogenetic abnormalities across plant species. Al^3+^ also exerts direct physicochemical effects that disrupt cell cycle progression independently of DDR signaling.

Within the nucleus, Al^3+^ ions, often applied as AlCl_3_, can bind to chromatin and DNA, causing DNA condensation, impaired transcription, and reduced cell division [33,63]. In *Zea mays*, exposure to 50 µM AlCl_3_ delayed DNA replication and slowed cell division in root tip cells [64]. In *Hordeum vulgare*, AlCl_3_ at 5–60 µM increased the proportion of cells in G2/M while reducing S-phase frequency, indicating altered cell cycle progression [22]. High concentrations of AlCl_3_ (5–25 mM) in the Misr 3 cultivar of *Vicia faba* decreased the mitotic index and increased chromosomal abnormalities, with metaphase being the most sensitive stage [65]. Similarly, low AlCl_3_ concentrations (50 µM) in seedlings caused abnormal cell division patterns, disrupting genetic balance [21]. In sunflower (*Helianthus annuus* L.), exposure to 5–100 µM AlCl_3_ for 24–72 h led to a progressive decrease in mitotic index and cytogenetic alterations such as C-mitosis, anaphase bridges, and chromosome stickiness. Al-induced extrusion of nucleolar material into the cytoplasm may result from Al-mediated loss of selectivity in the nuclear pore complex (NPC) [66]. Reduced mitotic, in *Vicia faba* [51], and meiotic indices were also observed in rice (*Oryza sativa*) and persisted in *Zea mays* even after prolonged recovery [21,52]. Beyond direct DNA interactions, Al interferes with hormonal regulation. In alfalfa (*Medicago sativa* L.), exposure to 100 µM AlCl_3_ significantly lowered indole-3-acetic acid (IAA) levels in apical buds and root tips, impairing root growth through altered cell division, elongation, and differentiation [67]. By affecting both DNA integrity and hormonal balance, Al disrupts cell cycle progression and root development while also modulating signaling cascades triggered by other stresses [68].

Defects in cell cycle regulation caused by plant cell exposure to Al are summarized in Table 1 and depicted in Figure 1.

### 2.4. The Effect of Al on DNA Synthesis in Plant Cells

Plant exposure to Al^3+^ ions induces extensive DNA damage, including double-strand breaks, which activate DDR pathways mediated by SOG1, ATR, and ATM. In *Arabidopsis* seeds, 1.5 mM AlCl_3_ causes Al-induced DNA double-strand breaks that involve homology-dependent recombination repair (HR). Notably, CK2 controls the DDR pathway through phosphorylation of SOG1. Under CK2-inhibiting conditions, SOG1 cannot be phosphorylated by ATM, suggesting a role for CK2 in priming SOG1 for its activation by the ATM kinase under DNA-damaging conditions. Hence, the loss of DNA damage response component, SOG1, can partially alleviate growth inhibition caused by Al, highlighting a complex relationship between DNA repair and stress tolerance [69,70]. In barley (*Hordeum*-TILLING-University of Silesia) exposed to 25–75 μM of AlCl_3_, mutations in the *HvATR* gene impair DDR signaling, leading to DNA fragmentation in root meristem cells; however, these mutants exhibit unexpected tolerance to Al toxicity, retaining root growth despite accumulated DNA lesions. This paradox highlights that reduced DDR activation may mitigate Al-induced growth arrest [59].

Al can also stimulate oxidative stress, generating ROS via NADH peroxidase and NADPH oxidase, which exacerbate DNA damage and cell death, as shown in *Allium cepa* exposed to 800 μM of AlCl_3_ [71]. Similar to heavy metals, such as cadmium (Cd), Al can interfere with nucleic acid synthesis. One proposed mechanism is the increased rigidity of the DNA double helix upon Al binding, which hinders the strand separation required for replication. Severe inhibition of DNA synthesis has been observed within 16 to 24 h in *Catharanthus roseus* (L.) G. Don under 0.2–1.0 mM AlCl_3_ exposure [72]. Furthermore, Al interaction with DNA can induce a conformational shift from the canonical B-form to the left-handed Z-form, increasing helical stiffness and making the DNA more resistant to unwinding. This structural change further inhibits replication and transcription processes [58,60].

DNA synthesis defects in plant cells due to Al exposure are summarized in Table 1 and graphically visualized in Figure 1.

### 2.5. Al Induction of Cell Death in Plants and Plant Cell Lines

Cell death in plants can be classified into programmed cell death (PCD) and accidental cell death, such as necrosis. PCD includes autophagic, apoptotic-like (AL-PCD), hypersensitive response (HR), and vacuolar (autolytic) types. While true apoptosis does not occur in plants, abiotic stressors, including Al, can trigger AL-PCD. PCD can also be categorized as developmental PCD (dPCD), regulated by internal stimuli, and environmental PCD (ePCD), triggered by external stressors like heavy metals [73]. The dPCD and ePCD have some similarities in morphological and biochemical features, such as calcium signaling, generation of ROS, or induction of vacuolar processing enzyme (VPE) activation, although the underlying gene regulation differs [74,75].

Al toxicity induces PCD primarily in root tips, where Al accumulates and disrupts cellular homeostasis. Evidence indicates that Al-induced PCD is mitochondria-dependent, characterized by chromatin condensation, DNA fragmentation, cytochrome c release, and activation of caspase-like proteases. ROS accumulation, mitochondrial membrane potential (ΔΨm) loss, and mitochondrial permeability transition pore (MPTP) further amplify the PCD cascade [76]. Comparative studies in peanut (*Arachis hypogaea*) root tip cells revealed that Al-sensitive genotypes exhibit faster and more intense PCD responses to 100 μM AlCl_3_ than Al-tolerant genotypes, establishing a negative correlation between PCD intensity and Al tolerance. AlCl_3_ exposure caused mitochondria-dependent apoptosis in a time- and dose-dependent manner, which was characterized by DNA cleavage, typical apoptotic chromatin condensation, apoptosis-related gene *Hrs203j* up-regulation, and cytochrome C release from mitochondria to cytosol, by several caspase-like proteases, and through the overexpression of metacaspase AhMC1 [77,78,79]. In parallel, studies in transgenic tobacco expressing the *Caenorhabditis elegans* apoptotic suppressor Ced-9 demonstrated that inhibition of Al-induced PCD and suppression of caspase-like vacuolar processing enzyme (VPE) activity enhanced root growth and reduced Al accumulation [80]. In *Arabidopsis*, Al inhibits the electron transport chain, leading to ROS accumulation, followed by mitochondrial membrane rupture and cytochrome C release into the cytosol. Overexpression of alternative oxidase 1a in mitochondria alleviated Al-induced PCD by preserving mitochondrial function [81]. Moreover, activity of caspase-3-, caspase-8-, and caspase-9-like proteins was also increased in *Triticum aestivum* root tip cells after exposure to Al_2_O_3_ nanoparticles (NPs) (5–50 mg mL^−1^) [82]. AlCl_3_ exposure caused cell death in tobacco cell lines BY-2 through impaired plasma membrane integrity induced by iron-mediated lipid peroxidation, mitochondrial dysfunction accompanied by ROS production, and Al-induced up-regulation of NtVPE1 encoding a VPE responsible for vacuolar collapse and plasma membrane integrity failure [10,83], and the cell death was apoptosis-like as it was accompanied by cell shrinkage and DNA fragmentation. Al, in the form of Al_2_O_3_ NPs in tobacco cell line BY-2, induced PCD through enhancement in ROS and NO production and executed through caspase-3-like enzyme activation in a time- and dose-dependent manner [84].

Al-induced PCD has also been reported across multiple plant species, including barley, wheat, maize, triticale, rye, oat, and peanut, with doses ranging from 0.1 μM to 50 mM AlCl_3_. Interestingly, low to moderate Al levels (0.1–1 mM AlCl_3_) typically trigger PCD, whereas high doses (10–50 mM AlCl_3_) induce necrosis [85,86]. The type of cell death depends on genotype, species, and stress severity, with low, moderate, and high stress inducing repair, PCD, and necrosis, respectively [87].

In addition to apoptosis-like PCD, plants exhibit vacuolar or autolytic PCD, marked by lytic vacuole accumulation, autophagosome formation, cytoplasmic shrinkage, intact organelles, and maintained turgor. Autophagy, a form of autolytic PCD, is crucial for nutrient remobilization, clearance of damaged organelles, and pathogen defense [88]. Autophagy can also mitigate Al toxicity, as reported in wheat (*Triticum aestivum* L.) root cells exposed to 30 μM AlCl_3_, where it efficiently removed damage and toxic macromolecules [89].

Al-induced cell death characteristics in plants and plant cell lines are summarized in Table 1 and visualized in Figure 1.

**Table 1:** Aluminum (Al)-induced genotoxicity associated with the formation of chromosome abnormalities, damage to the cytoskeleton, cell cycle, DNA synthesis, and cell death in plant tissues.

Species	Tissue	Al Compound	Al Dose	Al Duration	Biological Effect	Reference
Chromosome abnormalities						
*Pinus massoniana*		AlCl_3_	10^−5^–10^−2^ M	40 days	Nucleoproteins alteration, chromosomal aberrations, and cell division disruptions. Fragmentation and dispersion of NORs.	[18]
*Vicia faba*	Root	Al_2_(SO_4_)_3_	50 and 100 μM	72 h	Decrease in mitotic index. Induction of c-mitosis, chromosome stickiness, lagging chromosomes, and chromosome bridges in root tip cells. Alteration of NORs	[19]
*Vicia faba*	Root	AlCl_3_	0.01–10 mM	12 h	Increase in the frequencies of micronuclei and anaphase chromosome aberrations, decrease in the number of mitotic cells.	[24]
*Oryza sativa*	Root	AlCl_3_	50 μM	24 h	Chromosomal stickiness, micronuclei formation, laggards, and multinucleated cells. Inhibition of cell plate formation. Pollen sterility.	[21]
*Hordeum vulgare*	Root	AlCl_3_	5–60 μM	48–144 h	Reduction in mitotic activity of the root tip cells, induction of micronuclei, increase in the frequency of cells in G2/M phase.	[22]
*Zea mays*	Root	AlCl_3_	150, 300 and 450 μM	96 h	Lipid peroxidation, c-mitosis, micronuclei, bi- and trinucleated cells, starch reduction, callose formation, lignin accumulation, and intracellular Ca^2+^ increase.	[23]
*Allium cepa*	Root		0.5, 5, and 50 μM	12 days or 24 h	Reduction in mitotic activity, induction of nucleolar alteration, an increase in ROS, altered antioxidant enzyme activities	[52]
*Allium sativum*	Root	AlCl_3_	10^−5^–10^−1^ M	24, 48, and 72 h	Growth inhibition, c-mitosis, anaphase bridges, and chromosome stickiness. Inhibition of Ca uptake	[53]
Plant cell cytoskeleton						
*Citrus grandis* and *Citrus sinensis*	Root	AlCl_3_	1.0 mM	18 weeks	Reduction in root total soluble proteins, disorders in cell wall and cytoskeleton metabolism, energy and carbohydrate metabolism, and signal transduction.	[29]
*Citrus grandis* and *Citrus sinensis*	Leaves	AlCl_3_	1.2 mM	18 weeks	Photosynthesis inhibition, reduction in total soluble proteins. ROS Detoxification of ROS and other toxic compounds, such as aldehydes, is necessary.	[30]
*Triticum turgidum*	Root	AlCl_3_	1 μM 20, 100, and 500 μM	35 min to 24 h 6 h	Delayed transformation of the interzonal microtubule system into a phagmoplast, atypical tubulin bundle organization. Disturbance of cytokinesis, formation of polyploid cells.	[31]
*Glycine max*	Root	AlCl_3_	0.1 to 50 µM	30 min	Enhancement in actin filament tension. Formation of nonhydrolyzable [Al^3+^-ADP] or [AI^3+^-ATP] complexes, interference with F-actin filament assembly, and disorganization of cytoskeletal structures.	[35]
*Zea mays*	Root	AlCl_3_	50 µM	1 to 5 h	Alteration in microtubule orientation in protodermal cells, a change from transverse to longitudinal division plane, and reduction in axial root growth.	[36]
*Triticum aestivum*	Root	AlCl_3_	5 to 20 μM and 100 μM CaC1_2_	30 min	Al-induced inhibition of Ca^2+^ absorption.	[39]
*Triticum aestivum*	Root	AlCl_3_	0, 5, and 20 μM and 100 μM CaC1_2_	7 h	Al^3+^ effectively blocks the Ca^2+^ channel.	[40]
*Triticum aestivum*	Root	AlCl_3_	80 and 400 μM	6, 12, and 24 h	Al^3+^ effect on actin filaments, fimbrin-like protein mRNA accumulation, and alterations of cytoskeletal function.	[42]
Plant cell cycle						
*Hordeum vulgare*	Root	AlCl_3_	25, 50, 75 μM	7 days	The *hvatr.g* mutant shows increased DNA damage without affecting mitotic activity or the cell cycle profile.	[59]
*Zea mays*	Root	Al^3+^	90 μM	5 h	Inhibition of root elongation. The primary target of Al is the distal part of the transition zone of the root apex.	[61]
*Zea mays*	Root	AlCl_3_	50 μM	2 h and 10 h	Inhibition of cell division, disruption of Ca^2+^ uptake, reorganization of microtubules in the inner cortex.	[62]
*Zea mays*	Root	AlCl_3_	50 μM	5–180 min	Fast change in cell patterning, inhibition of auxin transport.	[64]
*Medicago sativa*	Root	AlCl_3_	100 μM	1, 3, and 10 days	Deformed cell shapes and a shorter length of the meristematic zone in root tips. Significantly decreased the IAA concentration in apical buds and root tips.	[67]
*Hordeum vulgare*	Root	AlCl_3_	5–60 μM	48–144 h	Shift in cell cycle progression, increased G2/M phase frequency, reduced S-phase frequency.	[22]
*Vicia faba*	Root	AlCl_3_	5, 15 and 25 mM	6, 12 and 24 h	Decrease in mitotic index and DNA integrity, increased chromosomal abnormalities, micronuclei formation. Metaphase was the most sensitive stage to Al stress.	[65]
*Oryza sativa*	Root	AlCl_3_	50 μM	24 h	Abnormal cell division and genetic imbalance.	[21]
*Helianthus annuus*	Root	AlCl_3_	5, 50, and 100 μM	24, 48, and 72 h	Decreased mitotic index, c-mitosis, anaphase bridges, and chromosome stickiness. Translocation of nucleolin from nucleolus to nucleoplasm and cytoplasm.	[66]
*Glycine max*	Root	AlCl_3_	100 mM	30 min to 72 h	Al entered cells of the sensitive genotype and accumulated at nuclei in the meristematic region of the root tip.	[68]
DNA synthesis in plant cells						
*Arabidopsis thaliana*	Root	AlCl_3_	1 and 1.5 mM	12 h and 10 days	DNA double-strand breaks. The *sog1* mutant is highly sensitive to Al.	[69]
*Arabidopsis thaliana*	Root	AlCl_3_	0.5 mM and 1.5 mM	5 days and 2 days	CK2 controls the DDR pathway through phosphorylation of SOG1. Under CK2-inhibiting conditions, SOG1 cannot be phosphorylated by ATM under DNA-damaging conditions.	[70]
*Hordeum vulgare*	Root	AlCl_3_	25, 50, and 75 μM	7 days	Mutations in the HvATR gene impair DDR signaling, leading to DNA fragmentation in root meristem cells.	[59]
*Allium cepa*	Root	AlCl_3_	10 to 1000 μM 800 μM	3 h 3 and 6 h	DNA damage and cell death induced by ROS burst through cell wall-bound NADH peroxidase and plasma membrane-associated NADPH oxidase.	[71]
*Catharanthus roseus*	Root	AlCl_3_	0.2, 0.5, and 1 mM	3 days or 24 and 48 h	Changes in putrescine and spermidine concentrations, inhibition of the enzymatic activities of arginine decarboxylase and S-adenosylmethionine decarboxylase, inhibition of DNA synthesis.	[72]
Cell death in plants						
*Arachis hypogaea*	Root	AlCl_3_	20, 100, and 400 μM	4 days	ROS accumulation, loss of ΔΨm, and DNA fragmentation, induction of programmed cell death.	[76]
*Arachis hypogaea*	Root	AlCl_3_	20, 50, 100, 200, and 400 μM or 100 μM	24 h or 4, 8, and 12 h	Programmed cell death manifested by DNA cleavage, chromatin condensation, apoptosis-related gene Hrs203j expression, and cytochrome C. Activation of Caspase3-like protease.	[77]
*Arachis hypogaea*	Root	AlCl_3_	100 μM	4, 8, and 12 h	Activation of caspase-like proteases, induction of programmed cell death.	[78]
*Arachis hypogaea*	Root	AlCl_3_	100 μM	12 h	Metacaspase AhMC1, a positive factor in Al-induced programmed cell death.	[79]
*Nicotiana tabacum*	Root	AlCl_3_	100, 300, 500, and 1000 μM	14 days	Ced-9, a Bcl-2 homolog, significantly alleviated Al inhibition of root elongation, decreased Al accumulation in the root tip, and inhibited Al-induced gene expression.	[80]
*Nicotiana tabacum*	Root	AlCl_3_	25, 50, 100, and 150 μM	18 h	Integrity of the plasma membrane and growth capacity were decreased, and the activity of vacuolar processing enzyme (VPE) was increased. The expression of VPE genes (VPE-1a, VPE-1b) was significantly enhanced.	[83]
*Nicotiana tabacum*	cell suspension culture	Al_2_O_3_ nanoparticles	10, 20, 50, and 100 μg mL^−1^	12, 24, 36, 48, 72, and 96 h	Induction of the processes of programmed cell death. Changes in the permeability of the plasma membrane. The loss of mitochondrial potential, the enhancement of the caspase-like activity, and the fragmentation of DNA.	[84]
*Arabidopsis thaliana*	Mesophyll protoplast	AlCl_3_	0.5 mM	10 to 60 min	Complex I and III of the mitochondrial electron-transport chain might be the sources of Al-induced mitochondrial ROS through interaction between Al and iron-sulfur (Fe-S) protein.	[81]
*Triticum aestivum*	Root	AlCl_3_ nanoparticles	5, 25, and 50 mg mL^−1^	96 h	Decrease in mitotic index, c-mitosis, monopolar metaphase, and stickiness. Induction of caspase 3, 8, and 9-like activities and programmed cell death.	[82]
*Triticum aestivum*	Root	AlCl_3_	30 μM	24 h	Al upregulated the expression of autophagy-related genes and enhanced the formation of autophagosomes. Al triggers the accumulation of oxidized proteins.	[89]
*Hordeum vulgare*	Root	AlCl_3_	0.1–50 mM	8 h or 3–12 h	ROS burst, Al-induced programmed cell death, necrosis in root-tip cells.	[85]
*Zea mays* *Triticum aestivum* *Secale cereale* *Triticosecale wittmack* *Hordeum vulgare*	Root	AlCl_3_	100 μM	0.5, 1, 2, 3, 4, 5, 6, 7, 8 h	DNA fragmentation, showing that wheat and maize are more tolerant than triticale, rye, barley, and oat, in which the PCD is active 0.5 h after Al induction.	[86]

## 3. Al Exposure to Mammals

### 3.1. Al-Mediated Genotoxicity in Mammals and Mammalian Cell Lines (Chromosome Aberrations)

Following exposure to 100 µM AlCl_3_, Al accumulates in the perinuclear cytoplasm of mammalian V79 cells [90]. Biochemically, Al^3+^ interacts strongly with phosphate groups in DNA, which may underline its genotoxic and mutagenic potential [91]. DNA damage can manifest as strand breaks, DNA adducts, oxidative lesions, chromosomal aberrations (breaks, micronuclei, aneuploidy), and gene mutations. Cytotoxicity and changes in cell cycle progression play significant roles in the evaluation of genotoxicity [92,93]. As DNA damage is well recognized as a critical factor in cancer development and progression, Al exposure has been studied for its carcinogenic potential. For example, welders exposed to Al exhibited high levels of genetic damage in lymphocytes, as measured by micronucleus formation [94].

A key consequence of Al exposure is chromosome instability (CIN), which includes structural (deletions, inversions, translocations) and numerical (aneuploidy) chromosomal alterations. CIN is frequently observed in human cancers and solid tumors and is often caused by defective mitotic divisions [95,96]. Culturing V79 cells with AlCl_3_ at physiologically relevant concentrations increased DNA double-strand breaks (DSBs) in a dose-dependent manner, possibly due to perturbation of sister chromatid segregation during mitosis [90]. The successful repartitioning of the cell genome relies on the bipolarity of the mitotic spindle, which ensures segregation of sister chromatids to each daughter cell [97]. Al-exposed cells exhibited abnormal multipolar spindles and clustered extra centrosomes [90], which are compensatory mechanisms to maintain bipolarity but lead to higher rates of chromosome missegregation and aneuploidy. In vivo studies in Swiss albino mice showed that single or repeated intraperitoneal injections of Al acetate (Al(OH)_2_(CH_3_COO)) (50–150 mg/kg body weight) induced chromosomal aberrations in bone marrow cells, including chromatid breaks, isochromatid breaks, rings, fragments, and complex aberrations, in a dose- and time-dependent manner [98]. Both in vitro and in vivo studies show that exposure to AlCl_3_ can induce malignant transformation. Mouse mammary epithelial cells exposed to AlCl_3_ underwent tumorigenic transformation, while mice (nude, NOD-SCID, and immunocompetent) developed tumors and metastases, associated with a 2.3–3-fold increase in unique structural chromosomal rearrangements [99,100]. The Al-induced transformation of normal mammary epithelial cells into tumor cells was reported to occur through enhancement in DNA double-strand break (DSB) formation, and due to failure in DSB repair, they can lead to cell transformation through chromosomal rearrangements or other mutation types conferring proliferative advantage and/or causing genomic instability [100]. The repair of Al-induced DSBs has been reported to be intrinsically mutagenic and not reversible upon Al withdrawal [99]. Chromosomal aberrations and epigenetic changes resulting in CIN and micronuclei formation are common in mitotic cells exposed to Al. Missegregated chromosomes trapped in micronuclei experience histone modification changes, leading to persistent chromatin accessibility abnormalities and enhanced genomic instability [101].

**Figure 2:**
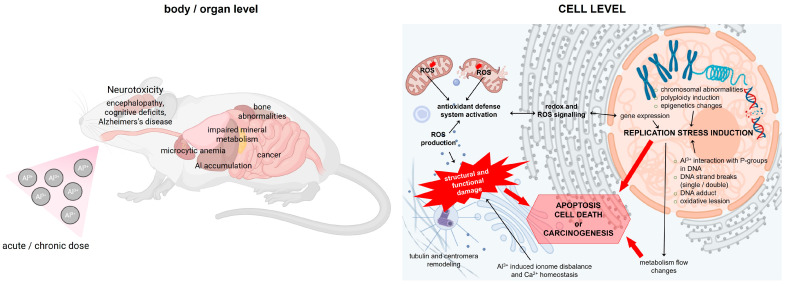
Mechanisms of Al-induced toxicity in mammalian cells. Exposure of mammalian cells to Al induces mitochondrial dysfunction, leading to excessive production of ROS and disruption of the antioxidant defense system, resulting in redox imbalance and impaired Ca^2+^ homeostasis. Al induces tubulin and centromere remodeling, leading to structural and functional damage of the cell. Al exposure is further associated with DNA damage, including replicative stress, DNA condensation, formation of Al–DNA adducts, and oxidative lesions. In addition, Al induces epigenetic alterations, chromosome abnormalities, and chromosome missegregation during cell division, which may result in polyploidy. Ultimately, Al-mediated inhibition of cell growth is linked to metabolic alterations that can culminate in cell death. In mammals, systemic Al exposure leads to Al accumulation in organs such as the liver, kidneys, bones, and brain, contributing to pathological conditions including encephalopathy, cognitive deficits, Alzheimer’s disease, microcytic anemia, bone disorders, and cancer.

Al-induced chromosome aberrations in mammals and mammalian cell lines are summarized in Table 2 and visualized in Figure 2.

### 3.2. Al Effects on Micronuclei Formation in Mammals

The micronucleus (MN) test is widely used to detect aneuploidogenic and clastogenic effects in mammalian cells. MNs are classified as centromere-positive, arising from chromosome missegregation or whole-chromosome loss, and centromere-negative, resulting from chromosome breakage and fragmentation. Repeated administration of AlCl_3_ in mammals increases MN formation in multiple organs, including the stomach, liver, and kidneys, and this increase correlates with elevated levels of apoptosis [102]. Both in vitro and in vivo studies show that Al exposure increases MN frequency in a dose-dependent or dose-independent manner. In Swiss mice, AlCl_3_ treatment increased MN formation in binucleated cells, with DNA damage levels positively correlating with MN frequency, supporting their use as biomarkers of Al-induced genotoxicity [102]. Similarly, oral administration of nano-Al_2_O_3_ to rats as three successive gavages at 6–25 mg/kg/bw resulted in increased DNA damage in bone marrow cells, as indicated by the comet assay. However, no increase in chromosomal mutations was observed [103]. Mechanistically, Al-induced MN formation has been linked to chromatin structural alterations, increased ROS production, lysosomal membrane destabilization, and DNase release into the nucleus [102,104]. Al treatment has been reported to increase MN in human lymphocytes through both clastogenic and aneuploidogenic mechanisms; however, the proportion of MN containing the whole chromosome was far higher, demonstrating its ability to effectively interfere with chromosome segregation [105].

Al-mediated micronuclei formation and DNA damage in mammals and mammalian cell lines are summarized in Table 2 and graphically visualized in Figure 2.

### 3.3. The Effects of Al Exposure on Cell Cycle Regulation in Mammalian Cells

Al disrupts cell cycle progression in mammalian cells by interfering with DNA synthesis, mitotic regulation, and chromosome segregation. In human lymphocytes, AlCl_3_ exposure at 5–25 µM reduced the mitotic index and induced DNA damage and chromosomal aberrations across all cell cycle phases, including G1, S, and G2 [106]. Structural and numerical chromosome abnormalities, polyploidy, and endoreduplication were observed, indicating that Al affects multiple components of the mitotic apparatus.

The increased frequency of chromosome breaks and gaps during the S phase is associated with the specific impact of AlCl_3_ on this phase, when DNA synthesis occurs. During the S phase, chromatin unwinds to allow for DNA replication, making the DNA more vulnerable to damage [107], including damage caused by toxic elements such as Al. Al salts, such as (Al(NO_3_)_3_), have the capability to relax the DNA totally and cause genetic malfunction through irreversible unwinding of DNA, contributing to replication stress and genetic instability [108]. Moreover, intracellular Al accumulation rapidly arrested proliferation in G2/M phase of the cell cycle as the percentage of cells in the G1 phase was reduced, and led to progressive augmentation of cells in the S and G2/M phases [90]. In the study involving human lymphocytes, AlCl_3_ caused an accumulation of cells in the S phase of the cell cycle. This accumulation may be due to a delay in the cell cycle or S phase arrest, which provides the cells with an opportunity to repair DNA damage. Additionally, Al exposure led to the formation of oxidized DNA bases. Pyrimidines were found to be less sensitive to Al-induced oxidation and/or were repaired more efficiently compared to purines, resulting in DNA breaks during the S phase [91]. Prolongation of the G_2_ phase favors the formation of multipolar spindles, a known source of aneuploidy and DNA double-strand breaks in missegregating chromosomes [109]. Interestingly, Al effects on mitosis are dose-dependent: low concentrations (2 µM AlCl_3_) can transiently stimulate mitotic activity, whereas higher doses inhibit mitosis, and this inhibition may be reversible [110].

Effects of Al exposure on cell cycle regulation in mammals are summarized in Table 2 and graphically depicted in Figure 2.

### 3.4. The Effects of Aluminum Exposure on Cell Proliferation and DNA Synthesis in Mammalian Cells

Cell proliferation and DNA synthesis are tightly linked to cell cycle regulation, and Al exposure disrupts this coordination in a cell-type- and dose-dependent manner. In human dermal fibroblasts, low concentrations of Al(NO_3_)_3_ stimulated DNA synthesis but delayed cell division, while higher concentrations and longer exposures inhibited proliferation. This unusual increase in DNA synthesis and not parallel-promoting mitosis was identified as a specific toxic effect of Al, ultimately leading to cell cycle arrest [111]. These biphasic effects are thought to be mediated through second messenger signaling pathways, with GTPase cycles as potential targets [112].

Cell-specific responses to Al have also been reported. In osteoprogenitor cells, AlCl_3_ promoted proliferation by inducing G_0_-S phase transition and increasing DNA synthesis and cell numbers [113]. In contrast, Al inhibited proliferation and DNA synthesis in MR 106-01 cells, shifting cellular activity toward matrix protein production [114]. Al can also impair DNA repair capacity by inhibiting zinc finger-containing proteins involved in DNA repair, thereby exacerbating genotoxic stress [115].

The chemical form of Al influences its cytotoxicity. In a study on cell proliferation and viability with Al salts, it has been found that only lipophilic aluminum acetylacetonate inhibited cell growth in neuroblastoma (SK-N-SH) and glioblastoma (T98G) cells, while non-lipophilic aluminum sulfate did not. Phosphate reduced the toxicity of aluminum acetylacetonate in T98G cells but did not prevent cell death in SK-N-SH cells, indicating that glial cells are more susceptible to Al toxicity than neuronal cells [116]. Lipophilic Al complexes, such as Al acetylacetonate, exhibit enhanced membrane permeability and intracellular accumulation, explaining their higher cytotoxicity compared with hydrophilic inorganic Al salts. This observation supports the notion that Al toxicity depends not only on pH or hydrolysis products, but also on Al speciation, solubility, and cellular accessibility.

Overall, Al effects on DNA synthesis and cell proliferation vary depending on cell type, Al concentration, chemical form, and culture conditions. These findings underscore the complexity of Al toxicity and highlight the need for further studies to define the mechanisms underlying their diverse cellular effects.

Effects of Al exposure on cell proliferation and DNA synthesis in mammalian cells are summarized in Table 2 and visualized in Figure 2.

**Table 2:** Al-induced genotoxicity associated with the formation of chromosome abnormalities, damage to the cytoskeleton, cell cycle, DNA synthesis, and cell death in mammal tissues and cell lines.

Species	Tissue	Al Compound	Al Dose	Al Duration	Biological Effect	Reference
Chromosome aberrations						
Mammalian	V79 cell culture HMEC cell culture	AlCl_3_	10, 100, 300, and 1000 μM	3 and 24 h	Accumulation of Al in the perinuclear region, an increase in DNA double-strand breaks, aneuploidy, and arrest in the G2/M phase of the cell cycle, formation of abnormal multipolar mitotic spindles that cluster supernumerary centrosomes.	[90]
Human	Human lymphocyte culture	AlCl_3_	1, 2, 5, 10, and 25 μg/mL	72 h	Induction of DNA damage, inhibition of DNA repair, and induction of apoptosis.	[91]
Human	Blood lymphocyte	Natural condition			Genetic damage in lymphocytes, and genetic polymorphisms of XRCC1 (p.Arg399Gln) and XRCC3 (p.Thr241Met).	[94]
*Mus musculus*	Albine mice	Aluminum acetate	50, 100, 150 mg/kg body weight, and 50 mg/kg weight	24 h 24, 48, and 72 h	Induction of chromosomal aberrations, reduction in mitotic index. Abnormal spermatozoa and reduction in sperm count. Increase in the frequency of micronucleated erythrocytes in the developing fetus.	[98]
*Mus musculus*	Mice and NMuMG cell culture	AlCl_3_	100 μM	14 days	NMuMG cells transformed in vitro by AlCl_3_ form large tumors and metastasize.	[99]
*Mus musculus*	HC11 and NMuMG cell culture	AlCl_3_	10 and 100 μM	71 weeks	Abnormal chromosomal structures in mammary epithelial cells. Genomic instability, without direct modulation of specific mRNA pathways.	[100]
Micronuclei formation						
*Mus musculus* Human	In vivo mice and in vitro human lymphocytes	AlCl_3_	49, 98, and 161 mg/kg body weight, and 5 μM, 10 μM, and 20 μM		An in vivo study showed an increase in micronuclei and irreversible lesions at all Al concentrations, indicating genotoxic potential and pathological disorders.	[102]
Rats	Male Sprague-Dawley	Al_2_O_3_ and Al_2_O_3_ nanoparticles and AlCl_3_	6, 12.5 and 25 mg/kg and 25 mg/kg body weight	24 and 45 h	DNA damage was analyzed in duodenum, liver, kidney, spleen, blood, and bone marrow, using alkaline and Fpg-modified comet assays. The assays revealed DNA damage from Al_2_O_3_ NPs in bone marrow, while AlCl_3_ caused slight, non-significant oxidative damage in blood.	[103]
Rats	Liver lysosomes	Al_2_(SO_4_)_3_ Al-glutamate, Al-acetylacetonate, AlF_3_	10 mM and 5 μM, 34 μM 75 μM		In the presence of Al^3+^, the proton pump activity is markedly reduced, compromising acidic vesicles functionality and lysosomal membrane destabilization.	[104]
Human	Human lymphocytes	Al_2_(SO_4_)_3_	0.5, 1, 2, and 4 mM	24 h	Clastogenic and aneuploidogenic induction of micronucleus formation.	[105]
Cell cycle						
Human	Human lymphocytes	AlCl_3_	5, 10, 15, and 25 μM	1 and 6 h	All tested Al concentrations were cytotoxic and reduced the mitotic index in all phases of the cell cycle. Al induced endoreduplication and polyploidy in treatments performed during the G1 phase.	[106]
Supercoiled pUC18	pUC18	Al(NO_3_)_3_	3.3 × 10^−7^, 1.6 × 10^−7^, 3.3 × 10^−8^ and 3.3 × 10^−9^ M		Al at physiologically relevant concentrations relaxed the intact supercoiled DNA and the topoisomers induced by chloroquine.	[108]
Human	Human lymphocyte culture	AlCl_3_	1, 2, 5, 10, and 25 μg/mL	72 h	Cell cycle restriction and DNA damage.	[91]
Human	whole blood cultures	AlCl_3_	2 and 4 mM	24 and 48 h	Low concentration transiently stimulates mitotic activity, whereas higher doses inhibit mitosis.	[110]
Cell proliferation and DNA synthesis						
*Mus musculus*	MC3T3-E1	AlCl_3_	1–50 μM	48 h	Al promoted proliferation by inducing G_0_-S phase transition and increasing DNA synthesis and cell numbers.	[113]
Rat	Osteoblast-like osteosarcoma cells UMR 106-01 line	AlCl_3_	100, 200, 400, and 1000 μg/L	24 h	Al depressed DNA synthesis, determined by 3H-thymidine incorporation. Al has an effect on cell division and protein synthesis.	[114]
Human	Glioblastoma T98G cell culture and neuroblastoma SK-N-SH cell culture	Al_2_(SO_4_)_3_ Al-acetylacetonate	0, 2, 10, 100, and 500 μM	48 h	Neuroblastoma cells are more susceptible to decreased cell proliferation, while the lipophilic Al salt is more toxic to glioblastoma cells.	[116]
Cell death						
Human	Human teratocarcinoma (NT2) precursor cells	Al-maltolate	5, 10, 50, 100, 250, and 500 μM	24 h and 48 h	Al maltolate at 500 μM cause cell death, induced chromatin condensation, nuclear fragmentation, apoptosis, and cytochrome c release.	[117]
*Mus musculus*	In vivo mice and in vitro human lymphocytes	AlCl_3_	49, 98, and 161 mg/kg body weight, and 5 μM, 10 μM, and 20 μM		Al shows genotoxic effects in vitro by causing DNA damage and indicates systemic toxicity in vivo, leading to morphological changes in the stomach, liver, and kidney.	[102]
Rat	Rat Wistar spleen lymphocytes	AlCl_3_	0.3, 0.6, 1.2 mM	24 h	Cyt c protein expression in cytoplasm, Caspase-3 and Caspase-9 activity, Bcl-2, Bax, Caspase-3 and Caspase-9 mRNA expressions, lymphocytes apoptosis index increased, while ΔΨm decreased.	[118]
*Mus musculus*	Neuro-2a neuroblastoma	AlCl_3_ Al-maltolate	1–1000 μM and 1–1500 μM	24 h	Al-malt increased cell death, resulting from a combination of apoptosis and necrosis, caspase 3 activation and the externalization of phosphatidyl serine. Nuclear condensation and fragmentation. Increase in p53 mRNA gene expression.	[119]
Rat	Astrocytes and neuron/astrocyte co-cultures	AlCl_3_	0.5, 1.0, or 2.0 mM	72 h	Morphological changes and apoptosis, cell shrinkage, aggregation, and fragmentation of chromatin, and formation of membrane-bound apoptotic bodies.	[120]
Human	SH-SY5Y human neuroblastoma	Al-maltolate	100, 200, 400, 500, and 600 μM	24 and 48 h	Increased ROS production and intracellular calcium levels, depletion of intracellular GSH levels, alterations in the levels of Nrf2, NQO1, pAKT, p21, Bax, Bcl2, Aβ1-40, and Cyt c.	[121]
Rat	PC12 cell culture	Al-maltolate	0.25, 0.5, 0.75, and 1 mM	48 h	Induction of apoptosis and an increase in MDA level and CAT activity. Downregulation of α-syn protein increased cell viability and decreased oxidative markers in Al treated cells.	[122]
Rat	PC12 cells culture	Al-maltolate	200 μM	24 h	Al triggers apoptosis and necroptosis in PC12 cells, up-regulates expressions of TNFR1, RIP1, and RIP3, and up-regulates expression of the phosphorylated mixed lineage kinase domain-like protein (MLKL).	[123]
Rat	PC12 cells culture	Al-maltolate	200 μM	24 h	Increased cell death and mitochondrial pathological changes in ferroptosis, inhibition of the cysteine/glutamate antiporter system, and depletion of glutathione.	[124]
Rat	In vivo	AlCl_3_	0, 50, 150, and 450 mg/kg	90 days	Impaired cognitive function, hippocampal lesions, oxidative stress, and altered expression of iron-related proteins were observed. AlCl_3_ causes iron dyshomeostasis, resulting in iron accumulation.	[125]

### 3.5. Al-Induced Cell Death in Mammal Cell Lines

Mammalian cell death is classified into two main types: necrosis and programmed cell death (PCD). Apoptosis, including classic apoptosis and anoikis, is the most common form of PCD, but there are also non-apoptotic forms such as autophagy, entosis, methuosis, paraptosis, mitoptosis, parthanatos, ferroptosis, pyroptosis, NETosis, necroptosis, etc. [126].

Apoptosis is a regulated process with key features like cell shrinkage, loss of contact with neighboring cells, decline in mitochondrial membrane potential, nucleus fragmentation, DNA cleavage into specific fragments, and formation of apoptotic bodies without inflammatory responses. In contrast, necrosis involves cell swelling, rupture, chromatin condensation, dysfunctional mitochondria, random DNA degradation, and increased inflammation [127].

Exposure to toxic substances can lead to cell death through necrosis or apoptosis. Genotoxicity from Al primarily results in programmed cell death (PCD), which helps prevent damaged genetic material from being passed on to future generations, thereby maintaining organism integrity. Al poisoning disrupts enzyme activity, alters protein synthesis and nucleic acid function, and affects cell membrane permeability. It can hinder DNA repair, destabilize DNA organization, increase ROS, induce oxidative stress, and disturb antioxidant enzyme activity. These changes can activate pathways like NF-kB, p53, and JNK, ultimately leading to apoptosis [128].

The experiments evaluating the toxicity of various Al compounds in different human and animal cell lines reported both cell apoptosis [117,129,130] and cell necrosis [102] as a response to Al treatment. The mechanisms involved in AlCl_3_-induced apoptosis in lymphocytes include an increase in Cyt C protein expression in the cytoplasm, Caspase 3 and Caspase 9 activity, caspase-3 and caspase-9 mRNA expressions, and the ratio of Bcl-2 and Bax mRNA expression, while mitochondrial transmembrane potential (ΔΨm) decreases [118]. In another study, exposure to aluminum maltolate (Al(mal)3) in vivo led to neurodegeneration characterized by the activation of Caspase 3 and the externalization of phosphatidylserine, both indicators of apoptosis. Additionally, nuclear condensation and fragmentation were observed. The type of cell death involved a combination of apoptosis and necrosis [119]. Exposure of astrocyte and neuron cells co-culture to AlCl_3_ caused both necrosis and apoptosis, but the intensity of apoptosis is much less compared with that of neurons, suggesting that astrocytes may be especially important for neuronal survival in the presence of Al [120]. AlCl_3_ in a concentration of 200 µM induced apoptosis in SH-SY5Y neuroblastoma cells by triggering endoplasmic reticulum stress and the production of ROS. This process subsequently disrupted the antioxidant defenses of neuronal cells through a pathway that is independent of p53 [121]. However, other studies reported that apoptosis in Neuro-2a cells is mediated through p53 signaling [119]. Al(mal)3-induced apoptosis in rat pheochromocytoma PC12 cells has been reported to be mediated through α-synuclein, a presynaptic protein expressed in the brain, which is involved in the regulation of neurotransmitter release, synaptic plasticity, and neuroprotection [122]. Another experiment with the PC12 cells demonstrated that AlCl_3_ can trigger both apoptosis and necroptosis. It was found to upregulate the expression of tumor necrosis factor receptor 1 (TNFR1) as well as receptor-interacting serine/threonine-protein kinases 1 (RIP1) and 3 (RIP3) at both the mRNA and protein levels. Notably, suppressing TNFR1 enhanced apoptosis while reducing the necroptosis-related expression of RIP1 and RIP3. Conversely, a deficiency in RIP1 and RIP3 resulted in a decrease in the extent of necroptosis [123]. Compared to apoptosis and autophagy, necroptosis appears to be the most prominent cell death pathway in Al-induced toxicity in neural cells and animal models for neurodegenerative diseases. Administration of necrostatin-1 (NEC-1) as a specific inhibitor of necroptosis reduced AlCl_3_-induced neuron apoptosis and phagocytosis through down-regulation of genes related to apoptosis and phagocytosis. Moreover, Al-induced necroptosis in SH-SY5Y cells has been reported to be mediated through Caspase 8 [131,132]. Necroptosis is a unique cell death type related to, but distinct from, both apoptosis and necrosis, characterized by a programmed necrotic cell death pathway and the activation of autophagy. Additionally, it has been suggested that necroptosis plays a role in Al-induced Alzheimer’s disease [132]. Al-mediated neurotoxicity may promote neurodegenerative processes in humans, as demonstrated in human NT2 cells, where Al(mal)3-induced cytochrome C release into the cytoplasm disrupted the mitochondrial membrane, leading to ROS production and oxidative stress [117].

Treating rat astrocytes with a low level of Al(mal)3 (400 µM) resulted in an upregulation of the autophagy-related protein Beclin 1. In contrast, at a high level of Al(mal)3 (1600 µM), there was an increase in the expression of both autophagy and apoptosis-related proteins. This pattern indicates that mild autophagy may occur before apoptosis at the lower Al dose, while at the higher dose, the significantly elevated autophagy may lead to cell apoptosis through the Beclin 1-dependent autophagy signaling pathway [133]. In fact, severe autophagy can induce apoptosis and autophagic apoptosis in neuronal cells and U2OS or Saos-2 cells [134,135]. Al(mal)3 has been reported to induce ferroptosis through activating the oxidative damage signaling pathway [124]. Ferroptosis is a type of cell death characterized by the accumulation of iron-based ROS and the generation of lipid peroxidation. Iron can initiate the redox reaction on its own, called Fenton reactions, and Al can promote and increase the severity of the reaction [136]. Oral administration of 50–450 mg/kg BW/d of AlCl_3_ for 90 days in drinking water induced the accumulation of iron, leading to oxidative damage in hippocampal neurons of rats [125].

Exposure to Al triggers a wide spectrum of regulated cell-death pathways, including oxidative-stress-mediated apoptosis, ferroptosis, autophagy-dependent death, pyroptosis, and necroptosis, underscoring the multifaceted nature of Al-induced toxicity. As the molecular mechanisms behind these forms of cell death become clearer [137], new opportunities arise to develop targeted interventions that limit cellular damage and improve outcomes in Al-related disorders. Future work translating these mechanistic insights into therapeutic strategies holds promise for reducing both the severity of Al toxicity and the side effects associated with current treatments.

Al-induced cell death characteristics in mammalian cells are summarized in Table 2.

## 4. Al Genotoxicity in Yeast Cells

Yeast cells are an excellent model organism in fundamental biological research due to many similarities in essential cellular processes with higher eukaryotes, including plants, animals, and humans. Moreover, due to easy manipulation and more controlled experimental conditions, rapid growth, and relatively low cost, yeast cells are preferred compared to other types of model organisms [138]. Several genes were identified to be involved in Al tolerance in yeast cells. The yeast cells knocked out for mitogen-activated protein kinase (MAPK) cascade genes, *slt2* or *bck1* (*slk1*), exhibit sensitivity to Al compared to the wild-type phenotype. This finding demonstrates that the MAPK signaling pathway is involved in the sensing and response to Al, contributing to Al tolerance [139]. In yeast, plant, and mammal cells, the MAPK family of proteins serves as central intracellular signal transducers that can regulate the transcription of different genes involved in osmo-adaptation, G1 and G2 cell cycle progression, and also general environmental stress protection during stress conditions [140,141,142].

Similar MAPK-dependent stress signaling frameworks operate in plants and mammals. In plants, MAPK cascades, composed of MAPKKKs, MAPKKs, and MAPKs linked by reversible phosphorylation, form central hubs that translate extracellular stress cues, including metal exposure, into transcriptional and physiological responses. Plant MAPKs are encoded by multigene families and integrate signals from ROS, phytohormones, and environmental stresses to regulate antioxidant defenses, growth, and development. Although MAPK cascades are not metal-specific, Al-induced ROS production has been shown to activate MAPKs, which then transmit metal stress signals to the nucleus, triggering adaptive responses; however, the precise downstream targets of MAPKs during metal stress remain incompletely defined [143,144,145]. Evidence from *Allium cepa* further suggests that MAPK signaling intersects with DNA repair networks during Al exposure, mediating both DNA damage at high Al^3+^ concentrations (800 μM) and adaptive genomic protection at low doses (10 μM) [146].

In mammals, MAPKs, including ERK, JNK, and p38, govern cell proliferation, differentiation, survival, and apoptosis, and Al exposure has been reported to decrease the activities of protein kinase C (PKC) and MAPK, particularly ERK signaling, leading to impaired neuronal plasticity, reduced kinase activity, and cognitive dysfunction of rats [147]. ERK has been associated with every major aspect of cell physiology. Notably, ERK signaling can exert both pro-survival and pro-death functions under metal-induced oxidative stress, depending on the intensity and duration of activation [148]. ERK connects key cellular components that link extracellular signals to the induction and activation of cell cycle events that control the transition from the G1 phase to the S phase, and it plays a role in the growth factor-independent G2/M phase of the cell cycle [149]. Affecting those cascades by stress, such as that caused by Al exposure, might result in cell cycle and severe cell proliferation defects. The *slt2* gene has been reported to play a role in cell cycle regulation in stress conditions [150]. Notably, the slt2 mutant of *Saccharomyces cerevisiae* (*S. cerevisiae*) was unable to cease cell division in the presence of toxic levels of Al. Additionally, the *slt2* gene encodes a MAPK kinase that plays a role in a signaling cascade downstream of protein kinase C (PKC). Therefore, PKC signaling is implicated in Al tolerance, as mutants in the PKC pathway show increased sensitivity to Al [139]. The PKC, as a member of the MAPK pathway, is involved in cell wall synthesis and cell cycle regulation during nutrient starvation, stress, and differentiation. In fact, this pathway is essential for arresting the cell cycle under stress conditions [151]. Collectively, these findings indicate that Al toxicity converges on MAPK-regulated pathways controlling cell cycle progression, stress adaptation, and survival across yeast, plants, and mammals, underscoring MAPKs as conserved mediators of Al-induced cellular stress and tolerance.

A genome-wide screening conducted in *S. cerevisiae* identified 37 genes associated with Al tolerance. These genes are involved in processes such as vesicle transport, signal transduction pathways, and protein mannosylation. Among the genes related to signal transduction, *cdc40*, *ptk2*, *sit4*, and *slt2* were highlighted, exhibiting molecular functions like nucleic acid binding, protein kinase activity, and protein serine/threonine phosphatase activity, respectively. The biological processes regulated by these genes include DNA replication, S phase of the mitotic cell cycle, assembly of the mitotic spindle (specific to fungi), nuclear mRNA splicing via the spliceosome, G1/S transition of the mitotic cell cycle, maintenance of cell ion homeostasis, polyamine transport, organization and biogenesis of the actin cytoskeleton, organization and biogenesis of the cell wall, protein amino acid phosphorylation, and protein kinase cascade [152].

From an interspecies perspective, the pre-mRNA processing factor CDC40 and the protein kinase Ptk2, which is associated with the regulation of polyamine transport and plasma membrane potential and was identified in a yeast whole-genome screen as contributing to aluminum (Al) tolerance, may play indirect roles in cellular adaptation to Al stress. This is consistent with the fact that global stress conditions frequently affect RNA processing, membrane homeostasis, and cell-cycle progression. Although CDC40 and Ptk2 are not canonical Al-tolerance determinants, investigating their potential contribution to Al stress responses in yeast, plants, and mammals represents an intriguing direction for future research and may help to identify conserved mechanisms underlying cellular adaptation to Al toxicity.

Additionally, the overexpression of genes responsible for magnesium transport, namely *alr1* and *alr2*, has been reported to enhance Al tolerance in *S. cerevisiae* [153]. Disruption of the *sso2* gene, which is part of the soluble N-ethylmaleimide-sensitive factor attachment protein receptor (SNARE) family, has been linked to increased sensitivity to Al. This increased sensitivity was associated with a higher content of unsaturated fatty acids in cellular lipids and an increase in ROS production in response to Al stress in the Δ*sso2* strain, compared to the wild type. This effect appears to occur not through the primary role of the gene in vesicle transport from the Golgi apparatus to the cell membrane but possibly due to the low Al levels in the culture medium, which may not have been sufficient to activate the vesicle transport system [154].

In plants, Al toxicity was shown to compromise intracellular Ca^2+^ homeostasis and block Ca^2+^ influx channels on the plasma membrane, whereas in *S. cerevisiae* cells, intracellular Ca^2+^ redistribution instead of extracellular Ca^2+^ influx may mediate Al toxicity. Expression of 6 mM AlCl_3_ for 6 h strongly impaired the intracellular Ca^2+^ homeostasis [155]. Additionally, vacuolar H^+^-ATPase (V-ATPase) has been shown to play a role in Al tolerance, as the Δ*vma1* and Δ*vma2* strains of *S. cerevisiae* exhibited a 40% reduction in growth compared to the control when exposed to 75 μM AlCl_3_ for 24 h [156]. In yeast cells, the vacuole serves as the principal Ca^2+^ sequestration site, which comprises more than 95% of cellular Ca^2+^ stores. This store is maintained mainly through the action of the high-affinity Ca^2+^ ATPase Pmc1p transporter. *S. cerevisiae* mutated cells lacking the vacuolar Pmc1p were shown to be sensitive to both Ca and Al toxicity induced by 0.2 M CaCl_2_ and 1.5 mM AlCl_3_, respectively [157]. Concomitant with this, the chemical-genomic profiling of the *S. cerevisiae* gene deletion collection showed that genes contributing to Al_2_(SO_4_)_3_ resistance were included in vacuolar transport, autophagy, cell wall macromolecule metabolic process, vesicle-mediated transport, and Ca^2+^ ion transport [158].

The administration of 10 mM Al^3+^ in the form of Al_2_(SO_4_)_3_ in *S. cerevisiae* culture media resulted in cell death affecting up to 38% of the cell population after 24 h of incubation. This cell death was linked to apoptosis triggered by an overproduction of ROS. In contrast, treating *S. cerevisiae* with low concentrations of Al (up to 2 mM) promoted cell division, while higher Al concentrations decreased cell division in a dose-dependent manner, depending on cell density. This suggests that there is a dynamic balance between Al-stimulated cell division and Al-induced cell death, with their overlapping effects ultimately determining whether the cells will grow or die [159]. Al at low concentrations has been reported to more actively participate in physiological processes besides its toxicity to cells, and other metal ion bioavailability would increase in response to low Al concentration [148], whereas in high concentrations, Al would restrict divalent cations uptake [160]. Al-induced cell death of *S. cerevisiae* was accompanied by cell shrinkage, vacuolation, chromatin marginalization, DNA degradation, nuclear fragmentation, DNA strand breaks, and abnormal cell aggregation as hallmarks of apoptosis. Rad1 is a protein involved in nucleotide excision repair of damaged DNA; its deletion, Rad1Δ mutant of *S. cerevisiae*, showed strong sensitivity to Al administration in culture medium, demonstrating Al-induced DNA damage [155]. In *S. cerevisiae*, AlCl_3_ (25 and 50 μg/mL) activated the antioxidant defense system. Following Al treatment, glutathione levels decreased, while superoxide dismutase and catalase activities increased. An oxidative stress sensitivity assay revealed a close link between Al-induced stress and glutathione. Notably, Al causes different oxidative damage compared to hydrogen peroxide, induces DNA damage, and its toxicity is related to sulfhydryl groups like glutathione, independent of YAP1, a transcription factor required for oxidative stress tolerance [161].

Proteomic analyses showed that 100 mM Al_2_(SO_4_)_3_ exposure to *Rhodotorula taiwanensis* RS1, a highly Al-tolerant yeast, reduced the abundance of proteins related to DNA transcription, protein translation, DNA defense, Golgi functions, and glucose metabolism. Moreover, MDH, one of the Al-responsive proteins, was induced under 100 mM Al and showed a strong correlation with increased enzyme activity and intracellular citrate levels. This suggests that MDH may serve as a valuable biomarker for Al tolerance [162]. In addition, mitochondrial function was also affected by Al_2_(SO_4_)_3_ exposure in *S. cerevisiae*, possibly due to increased ROS production and an affected antioxidant defense system, resulting in disrupted genome integrity and inhibited growth [163].

Sensitivity of proliferating cells to stimuli varies according to the phase of the cell cycle, and cells out of the stationary phase are more sensitive to environmental stresses. Administration of Al(OH)_3_ to the culture medium of *Schizosaccharomyces pombe* (*S. pombe*) yeast reduced cell growth in a dose-dependent manner, which was related to likely interactions of Al with intracellular components responsible for cell cycle and mitosis regulation. Furthermore, Al administration caused conformational changes in *S. pombe* cells, resulting in an increase in cell length and a decrease in cell width, as well as total cell destruction at high Al concentrations [164]. Al treatment of *Cryptococcus humicola*, an Al-tolerant yeast in acidic soil, reduced the size of cells and caused cell death in a dose-dependent manner [165]. Al is a commonly used coagulant for treating raw freshwater resources, as it effectively removes impurities and makes the water suitable for drinking. In contrast, as high Al concentrations are toxic for animals, yeast cells offer a viable option for removing heavy metals from food in the food processing industry, as well as from potable water during treatment. Certain strains of yeast, like Alt-OF2 and Alt-OF5, belonging to the genus Schizoblastosporion, are resistant to high concentrations of Al. These strains can absorb Al from their environment without negatively impacting their growth. This characteristic can be leveraged for the biological decontamination of heavy metals in various fields, including the food processing industry [166]. Conversely, a study with *S. pombe* cells showed that exposure of yeast cells to drinking water for 1 and 2 h significantly increased DNA damage, as indicated by the comet assay, compared to raw and distilled waters, which served as negative and laboratory controls, respectively [167].

**Figure 3:**
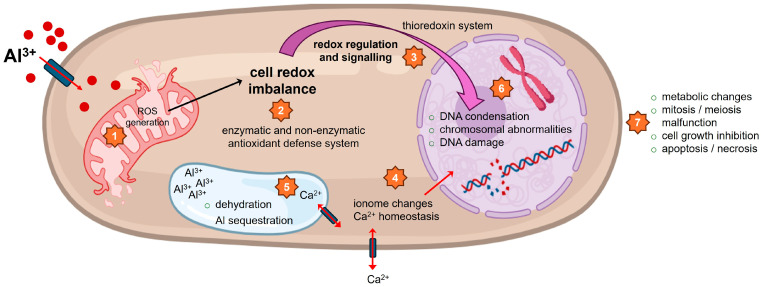
Mechanisms of Al-induced toxicity in yeast cells. Exposure of yeast cells to Al induces mitochondrial dysfunction (1), leading to excessive production of ROS and disruption of the antioxidant defense system, resulting in redox imbalance (2). Elevated oxidative stress alters ROS signaling pathways (3) and causes ionic imbalance, particularly through impairment of Ca^2+^ homeostasis (4). As a protective response, yeast cells sequester Al into the vacuole for detoxification, accompanied by Ca^2+^ release to mitigate Al toxicity (5). Al exposure is further associated with DNA condensation and damage, leading to chromosome abnormalities (6). Al-mediated chromosome missegregation during cell division disrupts cell cycle progression and affects mitosis and meiosis. Ultimately, Al-induced inhibition of cell growth is linked to metabolic alterations that may culminate in cell death (7).

The mechanisms underlying Al-mediated toxic effects that disrupt normal cellular homeostasis are illustrated in Figure 3.

Although many studies have evaluated the toxicity of Al on yeast cells, its genotoxicity requires further investigation to clarify the pathways involved. It is important to understand the mechanisms that control and preserve the cell genome, as well as the cell cycle regulation and proliferation, especially under environmental stresses such as Al toxicity. This knowledge could help in developing strategies to strengthen these preservation mechanisms. Additionally, yeast cells exhibit various types of cell death, including apoptosis, autophagy, and regulated necrosis, as part of their preservation strategies during stressful conditions. Further research is needed to explore the effects of Al-induced stress on the cell death responses of yeast.

## 5. Conclusions

The increasing exposure of organisms to Al, driven largely by human activities, raises significant concerns due to its widespread cytotoxic and genotoxic effects across biological systems. Although Al toxicity has been extensively studied in plants, mammals, and yeasts, major mechanistic gaps remain, particularly regarding how cellular damage is coordinated across the cytoskeleton, nucleus, chromatin, and cell cycle machinery. Emerging evidence highlights oxidative stress and DDRs as central mediators of Al toxicity in plants. However, the regulatory layers that fine-tune the balance between stress adaptation, growth arrest, and cell death remain largely unexplored. Notably, the role of non-coding RNAs, including microRNAs and long non-coding RNAs, in coordinating Al-responsive gene expression, chromatin dynamics, and stress signaling has received little attention. Addressing these gaps, especially the contribution of non-coding RNAs to Al-mediated cellular reprogramming, is essential for advancing a comprehensive understanding of Al toxicity and for identifying novel determinants of plant tolerance.

Although aluminum exposure is firmly associated with genotoxicity, chromosome instability, micronuclei formation, cell cycle dysregulation, and activation of diverse cell death pathways in mammalian cells, critical mechanistic gaps remain. In particular, the epigenetic consequences of Al-induced DNA damage, such as alterations in DNA methylation, histone modifications, chromatin accessibility, and higher-order genome organization, are still poorly characterized. While oxidative stress and defective DNA repair are recognized as major drivers of Al toxicity, little is known about how epigenetic remodeling integrates these signals to influence long-term genome stability, malignant transformation, or neurodegenerative outcomes. Addressing these epigenetic dimensions is essential for understanding the persistence, heritability, and disease relevance of Al-induced cellular damage.

Despite extensive use of yeast as a model for Al stress, several critical aspects of Al-mediated genotoxicity and cellular regulation remain insufficiently explored. While MAPK- and PKC-dependent signaling, vacuolar sequestration, and oxidative stress responses have been implicated in Al tolerance, the integration of these pathways with genome stability, chromatin organization, and cell cycle checkpoints is still poorly understood. In particular, the mechanistic links between Al-induced cytoskeletal perturbations, mitotic defects, and chromosome missegregation have not been systematically dissected, especially in *Schizosaccharomyces pombe*, where mitotic regulation more closely resembles that of higher eukaryotes.

Furthermore, although DNA damage and apoptosis-like cell death have been reported, the hierarchy and crosstalk between DNA repair pathways, redox signaling, TOR/MAPK signaling, and regulated cell death programs remain largely unresolved. Finally, most studies rely on acute or high-dose Al exposure, leaving the cellular consequences of chronic, low-dose Al stress, which are more environmentally relevant, largely unexplored. Addressing these gaps would significantly enhance the utility of yeast as a mechanistic model for aluminum toxicity and improve cross-kingdom comparisons with plants and mammals.

## Data Availability

No new data were created or analyzed in this study. Data sharing is not applicable to this article.
